# How to Measure the Reliability of Tasks in Experimental Psychology

**DOI:** 10.5334/joc.516

**Published:** 2026-08-18

**Authors:** Marc Brysbaert

**Affiliations:** 1 Department of Experimental Psychology, Ghent University, Belgium

**Keywords:** task reliability, R function, intraclass correlation coefficient

## Abstract

Calculating the reliability of experimental tasks can be surprisingly difficult using existing tools. Although R packages such as *psych* are robust, they often require data in wide format and assume carefully selected items that avoid floor and ceiling effects. To encourage the reporting of task reliability in experimental research, I have written an R function, *ICC_participants_long*, which uses the intraclass correlation coefficient (ICC) to measure the reliability of participant scores directly from data in long format. Applying this function revealed that the current split-half approach may underestimate the reliability of experimental tasks. Furthermore, the model-based approach makes it possible to generate Best Linear Unbiased Predictions (BLUPs) as estimates of participants’ scores, which provides an informative supplement to the raw means.

Experimental psychologists are increasingly interested in individual differences as a means of testing their theories (Brysbaert, 2024). In such research, they compare individual performance across different tasks, hoping not only to find significant correlations but also correlations strong enough to be of practical value. Often, however, the correlations turn out to be much weaker than hoped.

One reason for the low correlations between tasks is that the performance measure for the task is not reliable. Variables cannot correlate more strongly with each other than they do with themselves. Therefore, when we see correlations (or multiple regression analyses), we must ask ourselves how reliable the tasks are.

The R package psych (Revelle, 2026) is the standard package used by people working in the field of individual differences in psychology (but see Gamer, 2019, and Lüdecke, 2026, for other packages that have the same information). This package has its origins in intelligence and personality research, where the reliability of tests has been a major focus for a century. Unfortunately, this package often imposes fairly strict requirements on the data, which are difficult to meet in experimental psychology.

In recent years, a number of R packages for split-half correlation have been released in experimental psychology (Parsons, 2022; Pronk, 2025; Kahveci, 2026) that address some of the issues (particularly missing variables). Another package making use of linear mixed effects modeling is called psychometric (Fletcher, 2023).

In my experience, many researchers have difficulty with the existing software packages (which all require different input formats and use different default settings) and often use them incorrectly. That is why I decided to write a function called *ICC_participant_long*, which I believe is more user-friendly, provides the necessary information, and is designed to prevent common errors in data processing. The program also generates the Best Linear Unbiased Prediction (BLUP) for participant performance, which is an informative supplement to participants’ raw means (Haines, 2026). This text is intended to explain how to upload and use the function. It also provides useful background information about the output.

None of the techniques are new, and recent versions have benefited greatly from the flexibility offered by linear mixed-effects models (Baayen et al., 2008; Judd et al., 2012). I have simply combined a series of established steps so that the reliability of a task can be calculated with a single R command. This should provide useful information if you want to learn more about the reliability of a specific task for a specific group of participants.

## Datasets in which all participants provide data for all stimuli

The simplest analysis is when participants provide data for all stimuli. In that case, you can organize the data into a matrix similar to the one in Table 1. You have one row per participant and separate columns for the stimuli (trials). The example in Table 1 shows the accuracy data for 12 participants who completed 10 trials (for example, of a working memory task). You want to know how reliable the 10-item task is.

**Table 1:** Accuracy data of 12 participants on 10 items (wide format).


	STIM_1	STIM_2	STIM_3	STIM_4	STIM_5	STIM_6	STIM_7	STIM_8	STIM_9	STIM_10

**Part_01**	1	1	0	1	1	1	1	1	1	1

**Part_02**	1	1	0	1	1	1	1	0	1	1

**Part_03**	1	1	0	1	1	1	1	1	1	1

**Part_04**	0	0	0	0	1	1	0	0	1	1

**Part_05**	1	1	0	1	1	1	1	1	1	1

**Part_06**	0	1	0	1	1	1	0	1	1	1

**Part_07**	1	0	0	1	0	0	0	0	1	0

**Part_08**	1	1	0	1	0	1	1	0	1	1

**Part_09**	1	1	0	1	0	1	1	1	1	1

**Part_10**	1	1	0	1	1	1	1	0	1	1

**Part_11**	1	1	0	1	1	1	1	0	1	1

**Part_12**	1	0	0	1	1	1	0	0	1	0


The most frequent reliability measures used for this type of data are Cronbach alpha and McDonald omega. Both can be calculated with the R package *psych*. For alpha, the following code is used:


        
library(psych)
alpha(Table_1_wide)

      

This function returns the value of alpha = .75. We can get the omega coefficient with the following code:


        
omega(Table_1_wide)

      

Unfortunately, this function returns an error because there are two stimuli in Table 1 with no variance (Stim_3, which was incorrect for all participants, and Stim_9, which was correct for all participants). Omega cannot be calculated when some items show no variability. This is one of the reasons why researchers struggle with existing software packages, which were developed for research fields where much more attention is paid to the individual items (another origin of analysis problems is the presence of missing observations).

A more versatile reliability coefficient is the intraclass correlation coefficient (Shrout & Fleiss, 1979), because this is based on the variance components in an ANOVA or a linear mixed effects (LME) model. The following code is needed in *psych* to calculate ICCs:


        
ICC(Table_1_wide)

      

**Figure 1:**
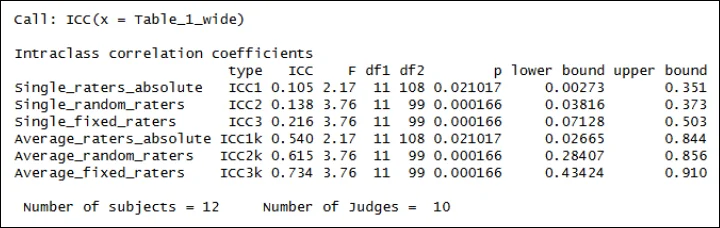
Output of the ICC() function in the R package psych, giving information about the intraclass correlation coefficients.

The procedure returns 6 rows of data, as shown in Figure 1.

The last three rows of the output are the most interesting.

ICC3k (consistency on items tested) is the equivalent of Cronbach’s alpha. It measures how consistently participants rank a specific set of stimuli, without taking into account how high or low the average scores of the participants are. It is typically the highest value (and therefore the most commonly reported). It is the standard measure for assessing the consistency of individual differences within a specific set of stimuli, as it focuses on the stability of a participant’s relative performance on that test.

ICC2k (interchangeable items agreement) uses a two-way random-effects model, in which both participants and stimuli are random samples from a larger population. ICC2k is more conservative than ICC3k because it evaluates absolute agreement; that is, whether the actual scores (not just rankings) remain consistent across items and participants. It answers the question: What value would you expect if you were to draw another sample of the same size from the stimulus pool? It is the model that will be used to calculate the BLUPs. The more homogeneous the stimuli are in your task, the closer ICC2k comes to ICC3k, since the average task score then becomes less dependent on the stimuli presented.

ICC1k (interchangeable observations agreement) is based on a one-way random-effects model and only accounts for variability between participants. This value is typically the lowest, because all variance arising from the stimuli (and their interaction with the participants) is treated as unexplained error. ICC1k treats all responses as interchangeable, effectively ignoring which stimulus produced which score. The more homogeneous the stimuli, the closer ICC1k will be to ICC3k. Conversely, when items vary in difficulty and the number of items is low, ICC1k will be significantly lower than ICC3k. As the number of items increases, the systematic variance of the stimuli averages out, causing ICC1k to converge toward ICC3k.

For readers familiar with linear mixed effects models, the following may be informative.

– ICC1k is based on the equation: lmer(Response ~ 1 + (1|Participant))
– ICC2k is based on the equation: lmer(Response ~ (1|Stimulus) + (1|Participant))
– ICC3k is based on the equation: lmer(Response ~ Stimulus + (1|Participant))

In each equation the variance explained by the random participant intercepts is compared to the residual variance (actually, to the variance consisting of the residual variance plus the participant variance). For ICC1, the residual variance includes all sources of error. This works when stimuli are interchangeable. ICC2 treats stimuli as a random effect, acknowledging that they are a sample from a larger population with their own random intercepts (so that some between-stimulus variance can be subtracted from the residual noise). The underlying model will be used to calculate the BLUPs. ICC3 treats stimuli as a fixed effect, allowing all between-stimulus variance to be subtracted from the residual variance. ICC3 assesses consistency across the specific set of stimuli tested, making it equivalent to Cronbach’s alpha. In this regard, it is interesting to know that Cronbach’s alpha largely corresponds to the average inter-item correlation (corrected for length attenuation). In inter-item correlations, pairwise correlations are calculated so that differences in means between items do not affect the value.

The “k” in the ICC name indicates that reliability is calculated based on the average of k observations per participant rather than on a single observation. Its operation is a generalization of Spearman-Brown formula that will be discussed below for correcting the split-half reliability, namely ICC_k = (k * ICC_) / (1 + (k-1) * ICC_). It explains the difference between first three lines of the *psych::ICC* output and the last three lines.

A remaining problem with the *psych::ICC* implementation is that it requires wide-format data, with which experimental psychologists are not familiar. Furthermore, some traditional psychometric implementations remove participants with missing observations listwise (i.e., all data of that participant are discarded), which poses a problem when the percentage of missing observations is quite high (e.g., reaction times on correct trials) or when many stimuli are presented per participant.

## Existing reliability software for long format input

Most experimental psychology software generates results in a long format. This differs from survey research, where results are typically displayed in a wide format (for example, by Qualtrics).

Each row in the long format contains a single observation and consists of at least three columns: participant, stimulus, and response, as shown in Table 2. The number, order, and names of the columns vary by software program. It is therefore useful to have a function that explicitly asks which column refers to what.

**Table 2:** Long format of Table 1 (contains 120 lines).


PARTICIPANT	STIMULUS	RESPONSE

Part_01	Stim_1	1

Part_02	Stim_1	1

Part_03	Stim_1	1

Part_04	Stim_1	0

Part_05	Stim_1	1

Part_06	Stim_1	0

Part_07	Stim_1	1

Part_08	Stim_1	1

Part_09	Stim_1	1

Part_10	Stim_1	1

Part_11	Stim_1	1

Part_12	Stim_1	1


I know of only two software packages that accept data in long-format and calculate ICC reliability. The first is *psychometric* (Fletcher, 2023). The following code is needed:


        
library(psychometric)
ICC2.lme(Response, Participant, data=Table_1_long)

      

The function yields a value of .54, which corresponds to ICC1k of *psych*. This value will work when all stimuli are expected to elicit the same response, but not when there are only a few stimuli that differ in difficulty.

The second package is *misty* (Yanagida, 2026). The code to use is:


        
library(misty)
multilevel.icc(Table_1_long, Response, cluster = “Participant”, type = “2”)

      

This provides the same value as *psychometric* (.54.). So, the output is equivalent to ICC1k (different from what the instruction type=”2” suggests).

Experimental psychologists have also developed their own R functions to calculate a task’s reliability. Interestingly, they have opted for the very first reliability index ever proposed: split-half reliability. The idea is simple: you divide the items into two halves, calculate the average performance on each half, and correlate the two scores (Kahveci et al., 2025). You must also apply a correction for the fact that the full test is more reliable than either of the halves. This is known as the Spearman-Brown correction for length attenuation and works as follows: the corrected correlation is equal to 2 * the observed correlation divided by 1 + the observed correlation. A correlation of 0.5 between the two halves of the test therefore translates to a split-half reliability of 2 * 0.5 / (1 + 0.5) = 0.67.

One appealing aspect of split-half reliability is that this method is robust to missing data; you do not need to have all observations to calculate the average performance in each half, as long as the missing observations are random. You simply calculate the means based on the available observations. Furthermore, modern computing power makes it possible to generate thousands of random splits, resulting in a stable distribution of correlations, rather than relying on a single, random split.

I know of three R packages that calculate split-half correlations (there are probably more).

The first package was presented by Pronk et al. (2022) and is called *splithalfr* (see also Pronk, 2025). This is the code:


        
library(splithalfr)
split_scores <- by_split(
  Table_1_long,
  Table_1_long$Participant,
  function(x) mean(x$Response, na.rm = TRUE),
  replications = 5000
)
coefs <- split_coefs(split_scores, spearman_brown)
mean_reliability <- mean(coefs, na.rm = TRUE)
print(mean_reliability)

      

The function *splithalfr* returns a single value, namely .555 (i.e., close to ICC1k). The function has a parameter match_participants, which can be changed from its default FALSE to TRUE.^1^ Then the splitting between variables no longer happens purely at random. When we run this, we get


        
split_scores <- by_split(
  Table_1_long,
  Table_1_long$Participant,
  function(x) mean(x$Response, na.rm = TRUE),
  match_participants = TRUE,
  replications = 5000
)
coefs <- split_coefs(split_scores, spearman_brown)
mean_reliability <- mean(coefs, na.rm = TRUE)
print(mean_reliability)

      

Now the function returns a value of .767, close to ICC3k.

The second package calculating split-half correlations is simply called *splithalf* (Parsons, 2022).


        
library(splithalf)
results <- splithalf(
  data = Table_1_long,
  outcome = “accuracy”,              # Logic for binary 0/1 data
  score = “average”,                 # Reliability of the mean accuracy
  var.participant = “Participant”,
  var.ACC = “Response”,              # Pointing to the accuracy column
  permutations = 5000,
  halftype = “random”
)
print(results$final_estimates)

      

It gives the following output:

**Figure:**
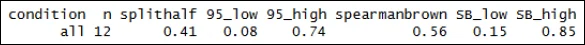


The split-half value (.56) is again similar to ICC1k.

The final package is *rapidsplithalf* (Kahveci, 2026), with the following instructions.


        
library(rapidsplithalf)
split_scores <- rapidsplit(data=Table_1_long,
  subjvar=“Participant”,
  stratvars=“Stimulus”,
  aggvar=“Response”,
  aggfunc=“means”,
  splits=5500
)
split_scores

      

The outcome says that the split-half reliability is .54:

**Figure:**
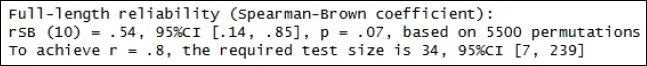


I admit that I was surprised to see that all split-half packages (by default) yield reliability estimates consistent with ICC1k, rather than ICC3k, as I had expected, given that Cronbach’s alpha corresponds to the mean of all the item correlations one can calculate in a table (corrected for length attenuation), and ICC3k is the intraclass correlation coefficient equivalent to Cronbach’s alpha.

In hindsight, it is not difficult to understand how the arbitrary division of items into two halves effectively results in items being treated as interchangeable (which corresponds to the model lmer(Response ~ 1 + (1|Participant))). This works well when the differences between the items are not thought to be important. Otherwise, you run the risk of underestimating the reliability of the task. The split-half correlation tacitly assumes that the two halves are matched on overall performance, not that they were taken at random. This is the reason why we see big difference in the package *splithalfr*, depending on whether the parameter *match_participants* is set to FALSE or TRUE. The difference will be particularly high for tasks involving a small number of items and when overall performance on the items varies.

## ICC_participant_long

Given the problems with the existing software and the fact that I developed a function to measure the reliability of stimulus norms based on intraclass correlation coefficients (Brysbaert, 2026), it seemed worthwhile to create an R function for task reliability that is easy to use and that attempts to avoid the pitfalls one encounters as a reviewer or editor. The function is called *ICC_participant_long*, because it is based on intraclass correlation coefficients calculated across participants and because it requires long-format input. It makes use of linear mixed effects models.

You can find the code for the *ICC_participant_long* function in the Appendix. To use it, save the code to a file in your working directory and name it “ICC_participant_long.R.” You can also download the file from https://osf.io/2sw5y/overview and place it in your working directory.

Then, all you need to do is to upload a long format data file that contains a column pointing to the participants, a column pointing to the stimuli, and a column pointing to the response (there can be more columns). In the example below, the dataset is called Table_1_long. Then use the following code:


        
source(“ICC_participant_long.R”)
my_iccs <- ICC_participant_long(
  participant = “Participant”,    # do not use participant = “participant”
  stimulus = “Stimulus”,          # do not use stimulus = “stimulus”
  response = “Response”,          # do not use response = “response”
  data = Table_1_long
)
print(my_iccs$ICCs)

      

The function asks you to specify which column names refer to the participants, the stimuli, and the responses. Hopefully, this explicit assignment will prevent users from accidentally swapping the columns for stimuli and participants. Make sure you use different names for your columns than the variable names in the function. Otherwise, this may create confusion in the calculations. So, do NOT use participant = “participant”, stimulus = “stimulus” or response = “response”. Use other names for your columns.

The program automatically codes participants and stimuli as factors, so no problems arise if numbers are used to refer to the participants or the stimuli. The program also asks you which column contains the dependent variable. This could be accuracy, reaction time, rating, or anything else that can be expressed as a number. The function automatically converts responses into numbers, since columns in R are often coded as characters when they contain missing observations.

The function will give you the following output:

**Figure:**
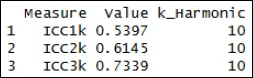


The function displays all three relevant ICCk measures, allowing you to see to what extent differences between items influence the reliability estimate. In addition, you will see the k_Harmonic measure, which indicates the number of observations (stimuli) over which the data are averaged. Since there were 10 stimuli per participant in the example and no observations were missing, the value is 10. If you compare, you will see that the output fully matches that of *psych*, as it should.

Now we can check what happens when we introduce two missing observations (randomly selected to be row 72 and 89). Then we get the following output:

**Figure:**
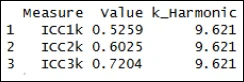


We have on average 9.62 observations per participant, but everything else remains very much the same.

I have included one final safeguard in the function. Many datasets contain outliers, resulting from input errors, data entry conventions (such as incorrect responses), or errors in data processing. We do not want such data to influence the output (including the BLUPs). Therefore, *ICC_participant_long* has a built-in outlier detector that rejects observations that deviate by more than 3 standard deviations (based on the residuals) from the value predicted by the item’s difficulty and the participant’s overall performance. This conservative threshold ensures that only extreme outliers are removed (Miller, 2023), no more than 1% for a good dataset. When outliers are detected and removed, a warning message is generated.

If you prefer to disable or weaken the outlier detection, you can set the outlier_sd to a high threshold (e.g., 10) as follows:


        
my_iccs_missing <- ICC_participant_long(
  participant = “Participant”,
  stimulus = “Stimulus”,
  response = “Response”,
  outlier_sd = 10,                        # increase SD distance
  data = Table_1_long_missing
)
print(my_iccs_missing$ICCs,digits=4)

      

Since the method of detecting outliers assumes a symmetric distribution, it may be worth considering transforming the dependent variable if it is strongly skewed. For example, reaction times (RT) are typically right-skewed. To achieve a more symmetric distribution, you can use a logarithmic transformation (log(RT)) or an inverse transformation (-1000/RT, representing information transmitted per second).

## Getting BLUPs for the participants

Another interesting aspect of the *ICC_participant_long* function is that it generates BLUPs (Best Linear Unbiased Predictions) for the participants. Researchers typically use mean scores as the best estimate for a participant. This is a good approach, but not the best possible, because it considers participants and stimuli in isolation. A better approach calculates the most likely estimate for a participant based on the full participant-by-stimulus matrix (Haines, 2026). This approach is particularly important in the case of missing observations (which may stem from difficult or easy items).

One way to calculate the BLUPs is to use the random participant intercepts in a mixed-effects analysis, in which both participants and stimuli are treated as random variables (i.e., the function underlying the ICC2k statistic). The participant BLUPs are stored in a distinct variable $Participants, which you can see with several commands, such as:


        
head(my_iccs$Participants,12)

      

This will show you the following output:

**Figure:**
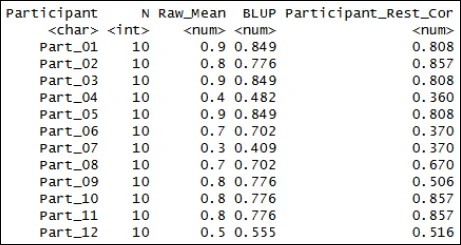


The first column is the participant. The second column (N) is the number of stimuli remaining after missing data and outliers have been excluded. The third column (Raw_Mean) shows the arithmetic mean for that specific participant. The fourth column shows the Best Linear Unbiased Prediction (BLUP) for the participant, taking into account the entire data matrix. Finally, the last column shows how much the participant’s scores correlate with the mean scores of the items based on the other participants. This is an effective way to identify careless participants, who will show low or negative correlations with the rest. If you want to obtain the same information for the stimuli, you can additionally run the *ICC_stimulus_long function* (Brysbaert, 2026).

If you look closely, you will notice shrinkage in the participant BLUPs: the highest raw means receive lower BLUPs, and the lowest raw means receive higher BLUPs. That is why BLUP estimates are generally better than raw means (extreme values are likely to show regression to the mean in a new study). There may be larger differences between BLUPs and raw averages when a significant number of observations are missing, because BLUPs are more robust to random data loss.

BLUPs are useful for research into individual differences because they provide the best estimate of an individual’s performance given a mixed-effects model and its assumptions. At the same time, one should be careful not to use them as dependent variables in secondary analyses, as these analyses assume independent observations, which BLUPs are not (Houslay & Wilson, 2017). So, it is not a good idea to calculate correlations between them or to run factor analysis on them. In such situations, it is better to jointly model all variables of interest in a single mixed-effects model, which properly accounts for dependencies (Houslay & Wilson, 2017; Kliegl et al., 2011; Rey-Sáez et al., 2026; Rouder et al., 2025, 2026).

You can easily save the BLUPs and the raw means in an Excel file with the code:


        
library(openxlsx)
write.xlsx(my_iccs$Participants,”outcome_analysis.xlsx”)

      

## ICC_participant_long_accuracy as an alternative for accuracy data

It is important to note that the ICCs in *ICC_participant_long* are based on a Gaussian model. This model works well for variables that are roughly normally distributed. Since we discussed an example of accuracy data in Table 1, an observant reader might wonder why I did not use a logit lme model (family = binomial) instead of a Gaussian model.

The first reason why I used the Gaussian approach is that it is in line with the usual practice of calculating Cronbach alpha, McDonald omega, and ICC. Indeed, in this way I was able to compare the outcome of my program with that of established packages, in particular *psych*. The second reason is that the Gaussian approach provides you with the reliability of mean accuracy, which is the variable you are most likely to use as the dependent variable in further analyses.

However, there is nothing to prevent us from using latent logit BLUPs instead of accuracy BLUPs as the best indicator of participants’ performance (De Boeck et al., 2011). When we do this, we assume that there is an underlying, normally distributed ability that determines whether or not participants will succeed on a particular item.

I adapted the *ICC_participant_long* function to an *ICC_participant_long_accuracy* function (see the second part of the Appendix). It requires accuracy data as input (only 0s and 1s) and calculates the reliability of latent participant scores. It also provides the best linear unbiased predictions (BLUPs) of the latent scores. The indices are based on the following models:

– ICC1k_latent = glmer(Response ~ 1 + (1|Participant), family = binomial)
– ICC2k_latent = glmer(Response ~ (1|Stimulus) + (1|Participant), family = binomial)
– ICC3k_latent = glmer(Response ~ Stimulus + (1|Participant), family = binomial)

Let us apply the procedure to our dataset:


        
source(“ICC_participant_long_accuracy.R”)
my_iccs_accuracy <- ICC_participant_long_accuracy(
  participant = “Participant”,
  stimulus = “Stimulus”,
  response = “Response”,
  data = Table_1_long
)
print(my_iccs_accuracy$ICCs,digits=4)

      

This gives:

**Figure:**
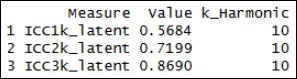


As expected, the values are slightly higher than the ICCs for the raw accuracy data. Importantly, you must keep in mind that they refer to the reliability of the latent variable, the normally distributed tendency or ability that is assumed behind the binary 0s and 1s. So, you cannot use your average accuracy scores and claim to have ICC_latent reliability. If you mention the ICC_latent reliability in your article, you must work with the BLUP_latent variables, which are logits (very similar to z-scores), calculated on the basis of the accuracy data.

You get the BLUP_latent scores as follows:


        
print(head(my_iccs_accuracy$Participants,12),digits=3)

      

**Figure:**
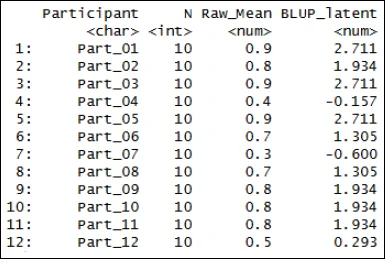


As noted earlier, you can use the BLUP scores to describe individual performance and to assess the extent to which the means correspond to the BLUPs in datasets with missing observations, but they cannot be used as dependent variables in secondary analyses, such as correlation tables or factor analysis, because they are not independent observations. For those analyses, raw means (or a transformation of them) can be used, as they are independent observations. It is also possible to include the raw data in a general analysis that encompasses all conditions (Houslay & Wilson, 2017; Rey-Sáez et al., 2026; Rouder et al., 2026).

The outlier function in the ICC_participant_long_accuracy function has also been simplified: it identifies any responses that are not 0 or 1 and removes them. This ensures that coding errors (like -999 for missing values) do not break the logistic model or bias your results.

## What about omega?

Readers may wonder how to reconcile the use of intraclass correlations (ICCs), which are related to Cronbach’s alpha, with the assertion that alpha is flawed and should be replaced by McDonald’s omega.

There are two key points to consider. First, most task-based researchers assume that their items measure a single underlying ability (unidimensionality). In such cases, the difference between alpha (ICC3k) and omega is typically negligible (Savalei & Reise, 2019; Savalei et al., 2019).

Second, much of the academic criticism of the alpha coefficient focuses on its use in “correcting for attenuation,” whereby an underestimation of reliability can lead to an overestimation of the correlation between two theoretical constructs. For most diagnostic and descriptive purposes, however, Cronbach’s alpha – and thus ICC3k – provides a very useful ballpark indication (Sijtsma & Pfadt, 2021). In practical research, the primary goal is to distinguish between a task with poor reliability (e.g., 0.30) and a task with good reliability (e.g., 0.70). This is different from the question of whether the “true” task reliability is 0.66 or 0.70. The functions described here are intended to provide a good rough estimate, also when your data does not meet all the requirements for an omega analysis.

## Going further

Creating functions to solve one problem usually raises questions about how to address related challenges. For example, readers may seek solutions for calculating the reliability of a difference score (e.g., the difference between congruent and incongruent trials in a Stroop task), or they may work with sparse datasets or multiple tasks/groups.

The *ICC_participant_long* and *ICC_participant_long_accuracy* functions are designed for single-task, single-group analyses. However, they can be extended or complemented in several ways. A useful strategy is to start with the simple functions described here and compare their output to the more complex alternatives. This approach helps you understand how each add-on affects your results and how to extract the right information from other packages.

### Difference scores

In theory, the LME approach can be extended to difference scores, but in practice it often performs poorly. Instead, it is better to use the R function developed by Kahveci et al. (2025; see also Kahveci, 2026), which is based on the split-half correlation of the effect size. This approach is robust because it accounts for all dependencies in the dataset through bootstrapping. For difference scores, the distinction between ICC1k and ICC2k is not an issue.

### Sparse datasets

For datasets with many missing values (e.g., different participants responding to different stimuli), it may be good to compare the output of *ICC_participant_long* with the function developed by Ten Hove et al. (2025). Their method provides more accurate (typically slightly lower) estimates of ICC2k and ICC3k for sparse data. Note that these functions do not screen for outliers or produce BLUP scores.

### Adding fixed and random effects

The LME models underlying the ICCk indices can be augmented with fixed and random effects known to influence the scores. For example, in RT experiments, RTs often decrease as participants become familiar with the task. To account for this, it is possible to include item order as a fixed effect and random slopes for item order over participants. This separates systematic practice effects from true individual differences, yielding more accurate and unbiased BLUPs (Baayen & Milin, 2010). Similarly, if participants belong to groups with known differences, it is possible to include group in the LME.

### Multiple tasks

For studies where participants complete several tasks, new analysis packages allow you to incorporate performance across tasks to improve estimates of individual ability and to correlate performance between tasks. Just as BLUPs leverage responses to multiple items, these approaches use data from multiple tasks to refine individual estimates. For further reading, see Rouder et al. (2025, 2026) and Rey-Sáez et al. (2026).

## Conclusion

In this article, I described how you can measure the reliability of a task you used. I pointed to several packages you can use and presented a new user-friendly function. If you use intraclass correlations coefficients, the following may be a good way to summarize your results.

We evaluated the reliability of our task using intraclass correlation coefficients (ICCs), which measure consistency in scores. The results showed that our stimuli generated consistent scores (ICC3k = .734, analogous to Cronbach’s alpha). Absolute agreement was lower (ICC1k = .540), indicating that the stimuli differed in average scores. Finally, the general reliability of the task (ICC2k = .614) suggests that if we were to use a different, yet similar, set of stimuli, the results would likely remain reasonably consistent.

## Additional File

The additional file for this article can be found as follows:

10.5334/joc.516.s1 Appendix. Code for functions ICC_participant_long and ICC_participant_long_accuracy.

## Data Availability

Materials, data, and analysis code can be accessed at: https://osf.io/xcv6e/.

## Note

Wolf Vanpaemel pointed this out to me. It shows that packages are generally flexible, but that a fair amount of knowledge and scrutiny is required to use them correctly.

## References

[1] Baayen, R. H., Davidson, D. J., & Bates, D. (2008). Mixed-effects modeling with crossed random effects for subjects and items. Journal of Memory and Language, 59, 390–412. 10.1016/j.jml.2007.12.005

[2] Baayen, R. H., & Milin, P. (2010). Analyzing reaction times. International journal of psychological research, 3(2), 12–28. 10.21500/20112084.807

[3] Brysbaert, M. (2024). Designing and evaluating tasks to measure individual differences in experimental psychology: A tutorial. Cognitive Research: Principles and Implications, 9(1), 11. 10.1186/s41235-024-00540-2 38411837 PMC10899130

[4] Brysbaert, M. (2026). Calculating the reliability of word norms. https://www.researchgate.net/publication/404289654

[5] De Boeck, P., Bakker, M., Zwitser, R., Nivard, M., Hofman, A., Tuerlinckx, F., & Partchev, I. (2011). The estimation of item response models with the lmer function from the lme4 package in R. Journal of Statistical Software, 39, 1–28. 10.18637/jss.v039.i12

[6] Fletcher, T. D. (2023). psychometric: Applied Psychometric Theory. https://cran.r-project.org/web/packages/psychometric/index.html

[7] Gamer, M. (2019). Package ‘irr’. https://cran.r-project.org/web/packages/irr/irr.pdf

[8] Haines, N. (2026, March 15). How to Estimate a Mean, and What It Means for Science. https://haines-lab.com/post/how-to-estimate-a-mean-and-what-it-means-for-science/

[9] Houslay, T. M., & Wilson, A. J. (2017). Avoiding the misuse of BLUP in behavioural ecology. Behavioral Ecology, 28(4), 948–952. 10.1093/beheco/arx023 29622923 PMC5873244

[10] Judd, C. M., Westfall, J., & Kenny, D. A. (2012). Treating stimuli as a random factor in social psychology: A new and comprehensive solution to a pervasive but largely ignored problem. Journal of Personality and Social Psychology, 103(1), 54–69. 10.1037/a0028347 22612667

[11] Kahveci, S. (2026). rapidsplithalf: A Fast Permutation-Based Split-Half Reliability Algorithm. https://cran.r-project.org/web/packages/rapidsplithalf/index.html

[12] Kahveci, S., Bathke, A. C., & Blechert, J. (2025). Reaction-time task reliability is more accurately computed with permutation-based split-half correlations than with Cronbach’s alpha. Psychonomic Bulletin & Review, 32(2), 652–673. 10.3758/s13423-024-02597-y 39443394 PMC12000231

[13] Kliegl, R., Wei, P., Dambacher, M., Yan, M., & Zhou, X. (2011). Experimental effects and individual differences in linear mixed models: Estimating the relationship between spatial, object, and attraction effects in visual attention. Frontiers in Psychology, 1, 238. 10.3389/fpsyg.2010.00238 21833292 PMC3153842

[14] Lüdecke, D. (2026). Package {performance}. https://cran.r-project.org/web//packages/performance/refman/performance.html#item_reliability

[15] Miller, J. (2023). Outlier exclusion procedures for reaction time analysis: The cures are generally worse than the disease. Journal of Experimental Psychology: General, 152(11), 3189. 10.1037/xge0001450 37498697

[16] Parsons, S. (2022). splithalf: Calculate Task Split Half Reliability Estimates. https://cran.r-project.org/web/packages/splithalf/index.html

[17] Pronk, T. (2025). splithalfr: Estimate Split-Half Reliabilities. https://cran.r-project.org/web/packages/splithalfr/index.html

[18] Pronk, T., Molenaar, D., Wiers, R. W., & Murre, J. (2022). Methods to split cognitive task data for estimating split-half reliability: A comprehensive review and systematic assessment. Psychonomic Bulletin & Review, 29(1), 44–54. 10.3758/s13423-021-01948-3 34100223 PMC8858277

[19] Revelle, W. (2026). psych: Procedures for Psychological, Psychometric, and Personality Research. https://cran.r-project.org/web/packages/psych/index.html

[20] Rey-Sáez, R., Franco-Martínez, A., Revuelta, J., & Vadillo, M. A. (2026). A unified framework for psychometrics in experimental psychology: The standardized generalized hierarchical factor model. Psychological Methods. Advance online publication. 10.1037/met0000846 42275019

[21] Rouder, J. N., Mehrvarz, M., & Schnuerch, M. (2026). The role of reliability in experiments. British Journal of Mathematical and Statistical Psychology, 1–15. 10.1111/bmsp.70042 41834697

[22] Rouder, J. N., Mehrvarz, M., & Stevenson, N. (2025). Hierarchical factor models for analysis in experimental designs. OSF. 10.31234/osf.io/cetwz_v3

[23] Savalei, V., Chen, B., & Vanpaemel, W. (2019). Break Coefficient Alpha! (Version 1.0) [Shiny app]. https://semlab.shinyapps.io/breakalpha/

[24] Savalei, V., & Reise, S. P. (2019). Don’t forget the model in your model-based reliability coefficients: A reply to McNeish. Collabra: Psychology, 5(1), 36. 10.1525/collabra.247

[25] Shrout, P. E., & Fleiss, J. L. (1979). Intraclass correlations: Uses in assessing rater reliability. Psychological Bulletin, 86(2), 420–428. 10.1037/0033-2909.86.2.420 18839484

[26] Sijtsma, K., & Pfadt, J. M. (2021). Part II: On the use, the misuse, and the very limited usefulness of Cronbach’s alpha: Discussing lower bounds and correlated errors. Psychometrika, 86(4), 843–860. 10.1007/s11336-021-09789-8 34387809 PMC8636457

[27] Ten Hove, D., Jorgensen, T. D., & Van der Ark, L. A. (2025). How to estimate intraclass correlation coefficients for interrater reliability from planned incomplete data. Multivariate Behavioral Research, 60(5), 1042–1061. 10.1080/00273171.2025.2507745 40524384

[28] Yanagida, T. (2026). misty: Miscellaneous Functions. https://cran.r-project.org/web/packages/misty/index.html

