# Supplementary material for: How to Measure the Reliability of Tasks in Experimental Psychology
Source: J Cogn. 2026 Aug 18;9(1):42. doi: 10.5334/joc.516 (PMC13496227; doi:10.5334/joc.516)
Supplement: Supplementary file 1 [file joc-9-1-516-s1.pdf]

Appendix:

Code for function ICC_participant_long

Copy and paste the code below into R and save it as the file ICC_participant_long.R file (or download the file from https://osf.io/2sw5y/files/osfstorage.

```
# ============================================================
# Function: ICC_participant_long
# Purpose: Calculates ICCs of tasks and provides BLUPs for participants
# Requires input in long format
# Usage:
#   source("ICC_stimulus_long.R")
#   results <- ICC_stimulus_long("SubjectID", "ItemID", "Score", my_df, outlier_sd)
# ============================================================


ICC_participant_long <- function(participant, stimulus, response, data, outlier_sd = 3) {
  if (!require("lme4")) {
    stop("The 'lme4' package is required but not installed. Please install it using:
install.packages('lme4')")
  }
  if (!require("data.table")) {
    stop("The 'data.table' package is required but not installed. Please install it using:
install.packages('data.table')")
  }

  # Check for reserved column names
  reserved_names <- c("participant", "stimulus", "response")
  if (participant %in% reserved_names || stimulus %in% reserved_names || response %in%
reserved_names) {
    stop(
      "Error: The column names 'participant', 'stimulus', and 'response' are reserved and cannot be
used. ",
      "Please rename these columns in your dataset to something else (e.g., 'Participant', 'Stimulus',
'Response')."
    )
  }

  library(lme4)
  library(data.table)

  # --- 1. Fast Data Prep ---
  dt <- as.data.table(data)
  dt <- dt[, .(P = as.factor(dt[[participant]]),
             S = as.factor(dt[[stimulus]]),
```

```
        R = as.numeric(as.character(dt[[response]]))]
dt <- dt[!is.na(R)]

# --- 2. High-Speed Outlier Detection (Expected Value Logic) ---
num_outliers <- 0
if (!is.null(outlier_sd)) {
  # Calculate components for expected value: E(y) = mu + alpha_i + beta_j
  dt[, mu_S := mean(R), by = S]
  dt[, mu_P := mean(R), by = P]
  grand_mu <- mean(dt$R)

  # Residual = Observed - (Stimulus_Mean + Participant_Mean - Grand_Mean)
  dt[, expected := mu_S + mu_P - grand_mu]
  dt[, resid := R - expected]

  sd_resid <- sd(dt$resid)
  outlier_idx <- abs(dt$resid) > (outlier_sd * sd_resid)
  num_outliers <- sum(outlier_idx)

  if (num_outliers > 0) {
    dt <- dt[!outlier_idx]
    message(paste("Removed", num_outliers, "outliers based on expected value residuals."))
  }
  # Clean up temp columns to save memory
  dt[, c("mu_S", "mu_P", "expected", "resid") := NULL]
}

# --- 3. Harmonic k ---
counts <- dt[, .N, by = P]$N
k_harm <- length(counts) / sum(1 / counts)

# --- 4. Single Model Optimization ---
tryCatch({
  # Two-way Random Effects Model
  m_main <- lmer(R ~ 1 + (1|S) + (1|P), data = dt)
  vc <- as.data.frame(lme4::VarCorr(m_main))

  v_S     <- vc$vcov[vc$grp == "S"]
  v_P     <- vc$vcov[vc$grp == "P"]
  v_resid <- vc$vcov[vc$grp == "Residual"]

  # One-way model is still needed for ICC1 specifically
  m_oneside <- lmer(R ~ 1 + (1|P), data = dt)
  v_P1      <- as.data.frame(lme4::VarCorr(m_oneside))$vcov[1]
  v_resid1  <- as.data.frame(lme4::VarCorr(m_oneside))$vcov[2]

  # --- 5. Calculate ICCs ---
```

```r
  # ICC(1,k): One-way random
  icc1k <- v_P1 / (v_P1 + (v_resid1 / k_harm))

  # ICC(2,k): Two-way random (Agreement)
  icc2k <- v_P / (v_P + (v_S + v_resid) / k_harm)

  # ICC(3,k): Two-way mixed (Consistency)
  icc3k <- v_P / (v_P + (v_resid / k_harm))

  # --- 6. Participant Scores and Participant-Rest Correlation ---
  grand_mean <- lme4::fixef(m_main)["(Intercept)"]
  blups      <- lme4::ranef(m_main)$P

  # Calculate counts and raw means
  part_stats <- dt[, .(N = as.integer(.N), Raw_Mean = mean(R)), by = .(Participant = P)]

  # Calculate Participant-Rest Correlation
  dt[, Stimulus_Sum := sum(R), by = S]
  dt[, Stimulus_Count := .N, by = S]
  dt[, Rest_Mean := (Stimulus_Sum - R) / (Stimulus_Count - 1)]
  dt[Stimulus_Count <= 1, Rest_Mean := NA]

  part_rest <- dt[!is.na(Rest_Mean), .(
    Participant_Rest_Cor = cor(R, Rest_Mean, use = "pairwise.complete.obs")
  ), by = .(Participant = P)]

  # Clean up temp columns
  dt[, c("Stimulus_Sum", "Stimulus_Count", "Rest_Mean") := NULL]

  # Create the BLUP table
  blup_table <- data.table(
    Participant = rownames(blups),
    BLUP = blups[,1] + grand_mean
  )

  # Merge all participant-level statistics
  final_participants <- merge(part_stats, blup_table, by = "Participant")
  final_participants <- merge(final_participants, part_rest, by = "Participant", all.x = TRUE)

  # Reorder columns
  setcolorder(final_participants, c("Participant", "N", "Raw_Mean", "BLUP",
"Participant_Rest_Cor"))

  return(list(
    ICCs = data.frame(
      Measure = c("ICC1k", "ICC2k", "ICC3k"),
      Value = c(icc1k, icc2k, icc3k),
```

```
      k_Harmonic = k_harm
    ),
    Participants = final_participants,
    Outliers_Removed = num_outliers
  ))

}, error = function(e) {
  stop("Model fitting failed: ", e$message)
})
}
```

Code for function ICC_participant_long_accuracy

Copy and paste the code below into R and save it as the file ICC_participant_long_accuracy.R file (or download the file from https://osf.io/2sw5y/files/osfstorage.

```
# ==============================================================
# Function: ICC_participant_long_accuracy
# Purpose: Calculates latent ICCs BLUPs for accuracy tasks
# Requires input in long format
# Usage:
#   source("ICC_stimulus_long_accuracy.R")
#   results <- ICC_stimulus_long("SubjectID", "ItemID", "Score", my_df)
# ==============================================================


ICC_participant_long_accuracy <- function(participant, stimulus, response, data) {
  if (!require("lme4")) {
    stop("The 'lme4' package is required but not installed. Please install it using:
install.packages('lme4')")
  }
  if (!require("data.table")) {
    stop("The 'data.table' package is required but not installed. Please install it using:
install.packages('data.table')")
  }
  library(lme4)
  library(data.table)

  # --- 1. Fast Data Prep ---
  dt <- as.data.table(data)
  dt <- dt[, .(P = as.factor(get(participant)),
             S = as.factor(get(stimulus)),
             R = as.numeric(as.character(get(response))))]
  dt <- dt[!is.na(R)]


  # --- 2. Clean Binary Data (Validation) ---
  num_outliers <- 0
  # Identify values that are not 0 or 1
  invalid_idx <- !(dt$R %in% c(0, 1))
  num_outliers <- sum(invalid_idx)

  if (num_outliers > 0) {
    dt <- dt[!invalid_idx]
    message(paste("Removed", num_outliers, "invalid observations (values other than 0 or 1)."))
```

```
}

# --- 3. Harmonic k ---
counts <- dt[, .N, by = P]$N
k_harm <- length(counts) / sum(1 / counts)

# --- 4. Single Model Optimization (Binomial Link) ---
tryCatch({
  # Define the theoretical logit residual variance inside the block
  # pi^2 / 3 is the variance of the standard logistic distribution
  v_resid_logit <- (pi^2) / 3

  # Two-way Random Effects Model (Logit)
  # Using nAGQ=0 can speed up glmer significantly if your dataset is huge
  m_main <- glmer(R ~ 1 + (1|S) + (1|P), data = dt, family = binomial)
  vc <- as.data.frame(VarCorr(m_main))

  v_S <- vc$vcov[vc$grp == "S"]
  v_P <- vc$vcov[vc$grp == "P"]

  # One-way model (Logit)
  m_oneside <- glmer(R ~ 1 + (1|P), data = dt, family = binomial)
  v_P1 <- as.data.frame(VarCorr(m_oneside))$vcov[1]

  # --- 5. Calculate ICCs (Latent Space) ---
  # ICC(1,k): One-way random
  icc1k <- v_P1 / (v_P1 + (v_resid_logit / k_harm))

  # ICC(2,k): Two-way random (Agreement)
  icc2k <- v_P / (v_P + (v_S + v_resid_logit) / k_harm)

  # ICC(3,k): Two-way mixed (Consistency)
  icc3k <- v_P / (v_P + (v_resid_logit / k_harm))


  # --- 6. Participant Scores ---
  grand_mean <- fixef(m_main)["(Intercept)"]
  blups      <- ranef(m_main)$P  # Correctly pulled P here

  # Calculate counts and raw means
  part_stats <- dt[, .(N = as.integer(.N), Raw_Mean = mean(R)), by = .(Participant = P)]

  # Create the BLUP table - FIX: Named the ID column 'Participant' to match part_stats
  blup_table <- data.table(
    Participant = rownames(blups),
```

```
    BLUP_latent = blups[,1] + grand_mean
  )

  # Merge and reorder columns
  final_participants <- merge(part_stats, blup_table, by = "Participant")
  setcolorder(final_participants, c("Participant", "N", "Raw_Mean", "BLUP_latent"))

  return(list(
    ICCs = data.frame(
      Measure = c("ICC1k_latent", "ICC2k_latent", "ICC3k_latent"),
      Value = c(icc1k, icc2k, icc3k),
      k_Harmonic = k_harm
    ),
    Participants = final_participants, # Renamed for clarity
    Outliers_Removed = num_outliers
  ))

 }, error = function(e) {
  stop("Model fitting failed: ", e$message)
 })
}
```
